# Application of JAK inhibitors in the treatment of rheumatoid arthritis: a systematic analysis based on clinical trial databases and registries

**DOI:** 10.3389/fmed.2026.1680115

**Published:** 2026-03-05

**Authors:** Junjie Cao, Rong Huang, Yanru Chen, Xu Li, Junyi Lou, Longyun Xu, Junxian Gu, Zining Luo, Tianliang Yao, Jiebin Xie

**Affiliations:** 1 Department of Gastrointestinal Surgery, Affiliated Hospital of North Sichuan Medical College, Nanchong, Sichuan Province, China; 2 School of Clinical Medicine, North Sichuan Medical College, Nanchong, Sichuan Province, China; 3 Medical School of Ophthalmology & Optometry, North Sichuan Medical College, Nanchong, Sichuan Province, China; 4 School of Basic Medical Sciences and Law, North Sichuan Medical College, Nanchong, Sichuan Province, China; 5 School of Medical Imaging, North Sichuan Medical College, Nanchong, Sichuan Province, China; 6 School of Stomatology, North Sichuan Medical College, Nanchong, Sichuan Province, China; 7 Department of Electronic Engineering, Faculty of Engineering, The Chinese University of Hong Kong, Hong Kong, Hong Kong SAR, China

**Keywords:** biological disease-modifying antirheumatic drugs, clinical trials, efficacy and safety, Janus kinase inhibitors, rheumatoid arthritis

## Abstract

**Objective:**

To systematically analyze 2014–2025 global clinical trial data of Janus kinase (JAK) inhibitors for rheumatoid arthritis (RA), clarify their R&D evolution, regional characteristics, efficacy and safety profiles, and provide evidence-based support for optimizing RA therapeutic strategies.

**Methods:**

Trials were retrieved from 16 international registries using PubMed MeSH and Embase Emtree terms, then screened per PRISMA guidelines for compliance with 2010 ACR/EULAR RA criteria and JAK-targeted therapy. A total of 87 eligible trials were analyzed for phase, molecular target, status, drug type and geographic region.

**Results:**

China (42 trials) and the U.S. (32 trials) dominated global research, with the U.S. leading R&D and China combining independent and collaborative studies. JAK2/JAK3-targeted trials rose from 25% (2014–2018) to 62.7% (2019–2025, *p* < 0.01), shifting toward multi-target combinations (e.g., TYK2 + JAK1) and away from Pan-JAK agents. JAK inhibitors showed target-dependent efficacy; infections were the most common adverse events, with serious adverse event (SAE) rates of 2.91–7.37% and drug-specific safety profiles. Trial data disclosure was uneven, with gaps in investigational drug and Phase IV trial data.

**Conclusion:**

JAK inhibitors are key RA therapeutics with target-distinct efficacy and safety. Future efforts should prioritize high-quality Phase IV studies, regional subgroup analyses, head-to-head target comparisons and standardized data disclosure.

**Systematic review registration:**

https://www.crd.york.ac.uk/PROSPERO/view/CRD420261290714, Identifier CRD420261290714.

## Introduction

Rheumatoid arthritis (RA) is a chronic, systemic autoimmune disease characterized primarily by erosive, symmetrical polyarthritis. In severe cases, it can lead to permanent joint damage, disability, and even death (1). Moreover, RA has systemic effects and may involve other organs, including the lungs, heart, blood vessels, skin, and eyes. In addition, a modeling study predicted that by 2050, approximately 3.17 million (95% CI: 2.58–3.90 million) people worldwide will be affected by rheumatoid arthritis, 68.7% of women (2). Currently, the exact pathogenesis of RA remains unclear, and there is no known cure. However, various treatment strategies are available, including pharmacological interventions—such as nonsteroidal anti-inflammatory drugs (NSAIDs), corticosteroids, conventional disease-modifying antirheumatic drugs (DMARDs), and biologic DMARDs (bDMARDs)—as well as physical exercise and rehabilitation therapies.

In recent years, with advances in understanding the molecular pathogenesis of RA and the intracellular signaling pathways involved in its development, targeted therapies have gained increasing attention. As research into the JAK-STAT signaling pathway has increased, a growing number of novel JAK inhibitors—such as momelotinib and pacritinib—have emerged. The efficacy of targeted synthetic disease-modifying antirheumatic drugs (tsDMARDs), particularly JAK inhibitors, is now comparable to that of biologic DMARDs (bDMARDs) such as tumor necrosis factor inhibitors (TNFis) (3). JAK inhibitors are a class of drugs used to treat various immune-related diseases by selectively inhibiting Janus kinases 1, 2, and 3 (JAK1, JAK2, and JAK3) and tyrosine kinase 2 (TYK2), thereby blocking the JAK-STAT signaling pathway, modulating immune responses, and alleviating inflammation and symptoms. To date, six JAK inhibitors—including tofacitinib, baricitinib, peficitinib, upadacitinib, filgotinib and ivarmacitinib sulfate—have been approved by different regulatory agencies for the treatment of RA. Notably, ivarmacitinib sulfate was recently approved in April 2025 by the National Medical Products Administration (NMPA) of China for RA treatment. In clinical practice, these JAK inhibitors have demonstrated significantly better treatment outcomes and responsiveness than conventional DMARDs (cDMARDs) (4, 5). In summary, this study aims to systematically evaluate the latest advancements in the use of JAK inhibitors for the treatment of RA, providing valuable insights to support the optimization of therapeutic strategies and the improvement of patient quality of life.

Based on the need to systematically synthesize the current status of clinical research on JAK inhibitors for rheumatoid arthritis (RA) and address the gap in integrated evidence, we aim to clarify the core directions of this field through targeted analysis. Therefore, the primary objective of this study is to systematically analyze the global clinical trial data of JAK inhibitors for RA spanning 2014–2025, and thereby clarify the overall landscape of their research and development (R&D) evolution, regional participation characteristics, and key efficacy and safety outcomes. The secondary objectives are as follows: (1) To explore the temporal changes in target strategies of JAK inhibitor development for RA, reflecting the dynamic adjustment of research focuses with advances in pathogenesis research; (2) To identify publication bias in trial result disclosure across different drug types, targets, and trial phases, so as to provide insights into the completeness of current evidence; (3) To further provide evidence-based references for optimizing clinical therapeutic strategies of RA and guiding the subsequent R&D of JAK inhibitors, bridging the gap between clinical trial findings and real-world practice.

## Method

**Figure 1:**
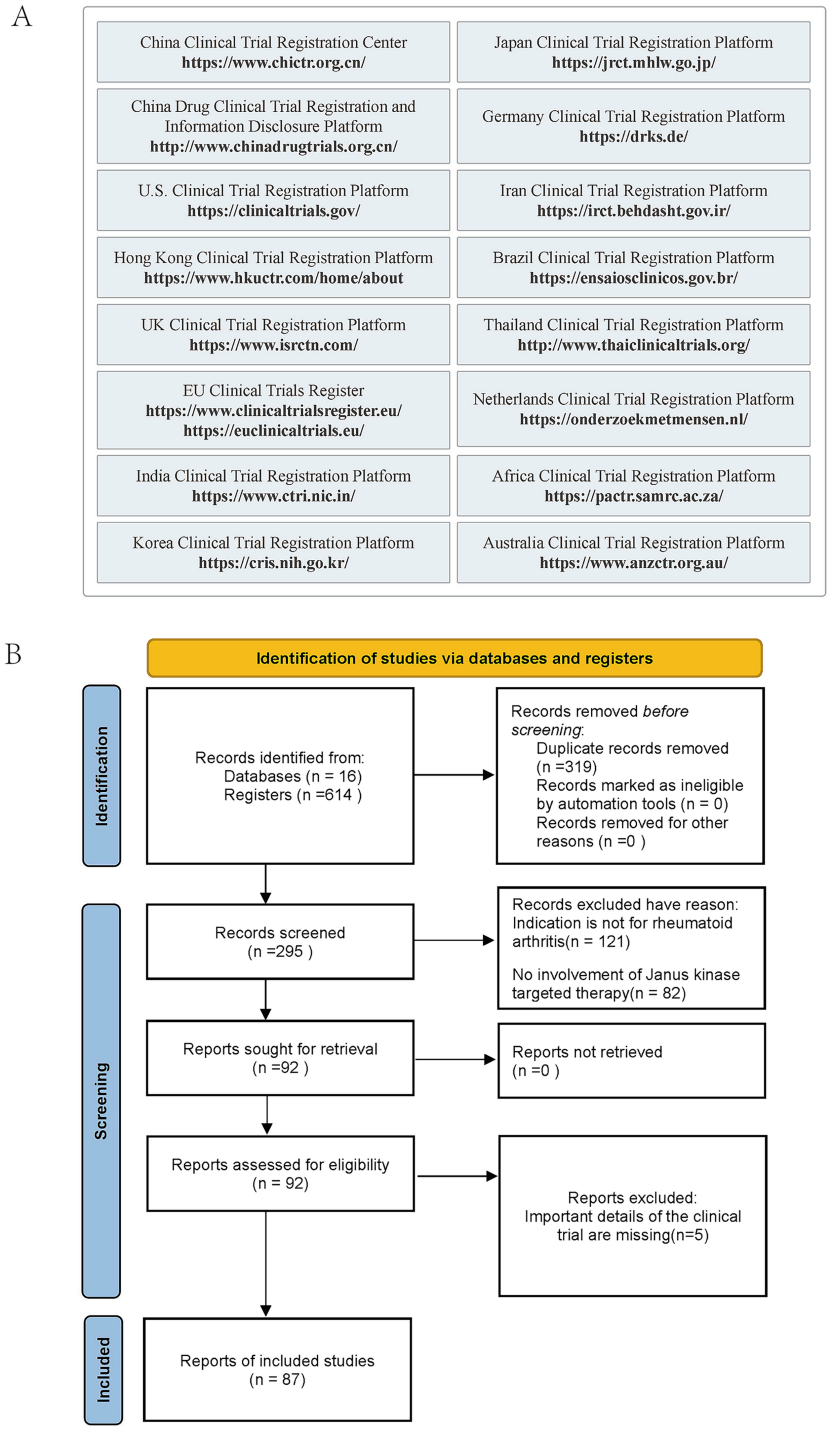
Clinical trial sources and inclusion and exclusion flowchart. **(A)** Clinical trial sources. **(B)** PRISMA-based clinical trial screening flowchart.

This study was registered in PROSPERO (CRD420261290714). By searching 16 clinical trial registries (Figure 1A), a systematic analysis was conducted on clinical trials related to arthritis and rheumatoid arthritis, with the time range limited to January 1, 2014 to April 5, 2025. Using controlled vocabulary terms from the PubMed MeSH and Embase Emtree systems, the search strategy incorporated terms such as “Janus kinase inhibitors,” “JAK inhibitor,” “Arthritis, Rheumatoid,” “rheumatism arthritis,” and “rheumatoid arthritis,” along with their synonyms and related terms, the complete search strings are: “(‘Arthritis, Rheumatoid’ OR ‘rheumatism arthritis’ OR ‘rheumatoid arthritis’) AND (‘Inhibitors, Janus Kinase’ OR ‘Kinase Inhibitors, Janus’ OR ‘Janus Kinase Inhibitor’ OR ‘Inhibitor, Janus Kinase’ OR ‘Kinase Inhibitor, Janus’ OR ‘JAK Inhibitor’ OR ‘Inhibitor, JAK’ OR ‘JAK Inhibitors’ OR ‘Inhibitors, JAK’).” The search characters for each clinical trial database can be found in Appendix 2. A total of 614 candidate studies were initially identified, the screening process was independently conducted by researchers Cao and Huang in accordance with the PRISMA statement (Figure 1B), with the specific procedure as follows:

1. Duplicate record elimination stage: Using the Excel management tool, duplicate records were identified and removed by comparing core information such as trial registration numbers, research teams, research institutions, sample sizes, patient baseline characteristics, and study designs. If the core information was highly overlapping but the trial registration number was missing, cross-validation of the study title and registration time was used to further determine whether the records were duplicates.
2. Initial screening based on title, indication, and drug: Preliminary screening was conducted based on the title, intervention measures, and disease type to exclude studies that were clearly not eligible for inclusion. The inclusion criteria were clearly defined as follows: ① The indication was limited to rheumatoid arthritis (RA) or the study subjects had to meet the classification criteria for rheumatoid arthritis jointly established by the American College of Rheumatology (ACR) and the European League Against Rheumatism (EULAR) in 2010 (6). ② The core evaluation content must involve Janus kinase (JAK) targeted therapy, whether as monotherapy or combination therapy. The JAK inhibitors included in the search are as follows: tofacitinib, baricitinib, peficitinib, upadacitinib, filgotinib, delgocitinib, ivarmacitinib sulfate, momelotinib, pacritinib, etc. (covering both approved and investigational JAK inhibitors targeting JAK1, JAK2, JAK3, TYK2, or multi-target combinations). ③ All trials that fully meet the aforementioned inclusion criteria are included in the analysis, regardless of their trial status (e.g., completed, recruiting, active but not recruiting, unknown status, etc.). The exclusion criteria are clearly defined as follows: ① The research topic is not related to RA or does not explicitly use the ACR/EULAR 2010 criteria; ② The indication covers other immune - related diseases besides RA (such as psoriatic arthritis); ③ Studies that do not involve JAK - targeted therapy and only evaluate other targets in the JAK pathway (such as STAT inhibitors) or non - targeted drugs (such as conventional DMARDs). ④ Important details of the clinical trial are missing (such as specific JAK-targeted drugs)
3. Full-text screening stage: For the studies that passed the initial screening stage, all information was obtained and read to further verify whether they fully met the inclusion criteria. The focus was on confirming the specific types of interventions (types of JAK inhibitors and dosing regimens), the purity of the study subjects’ indications (whether there were any subgroups of non-RA patients), and the completeness of the study design. Studies with incomplete information or implicit non-compliance with the inclusion criteria were excluded.

During the screening process, the two researchers independently recorded the number of included/excluded trials and the reasons for exclusion at each stage. In case of screening disagreements, the priority was to clarify the points of contention through face-to-face discussions and reach a consensus. If consensus could not be reached after discussion, a third researcher (YC) would arbitrate, and the final arbitration result would be the ultimate screening conclusion.

The key extracted and compared elements included the study initiation year and phase, molecular target, trial type, trial status, endpoint classification, drug name, and geographic region of the study.

### Publication bias analysis

This analysis aims to evaluate the differences in the disclosure of trial results among different subgroups. The sub-categories analysed include: (1) Drug level: comparing result disclosure between mature approved drugs and investigational drugs; (2) Target level: comparing result disclosure between single-target (e.g., JAK1) and multi-target (e.g., TYK2 + JAK1) JAK inhibitors; (3) Trial phase level: comparing result disclosure across Phase 0, I, II, III, IV, and Phase II/III trials. The disclosure status is determined based on whether the trial has publicly available primary or secondary outcome data in the retrieved databases/registries.

## Results

According to the statistics, the two evaluators made judgments on 614 projects regarding the inclusion criteria of the trial. Among them, 585 projects reached consensus (consensus on joint inclusion + consensus on joint exclusion), with a consistency rate of 95.3% (585/614). The distribution of the types of consensus is as follows: (1) Consensus on joint inclusion: A total of 62 trials were judged as “inclusion” by both evaluators A and B, accounting for 10.6% (62/585) of the total consensus; (2) Consensus on joint exclusion: A total of 523 trials were judged as “exclusion” by both evaluators, accounting for 89.4% (523/585) of the total consensus; (3) Disagreement in judgment: A total of 29 projects did not reach consensus (614–585), including 10 cases of “A judged inclusion, B judged exclusion” and 19 cases of “A judged exclusion, B judged inclusion.” Based on the above results, the Cohen’s Kappa coefficient was calculated as 0.73. According to the coefficient interpretation standard (0.61–0.80 indicates moderate to good reliability), it indicates that the two evaluators have moderate to good consistency in their understanding and implementation of the trial inclusion criteria.

The subsequent screening and exclusion process is as follows (Figure 1B): 121 trials were excluded due to not being for rheumatoid arthritis (*n* = 121); 82 trials were excluded because they did not involve Janus kinase targeted therapy (*n* = 82); After the team members further verified the detailed information of the trials, 4 trials were excluded because they lacked key research data. In total, this study determined 87 trials that met the inclusion criteria (see Appendix Table 1).

### International collaboration and population characteristics (Figures 2A,B and Tables 1, 2)

**Figure 2:**
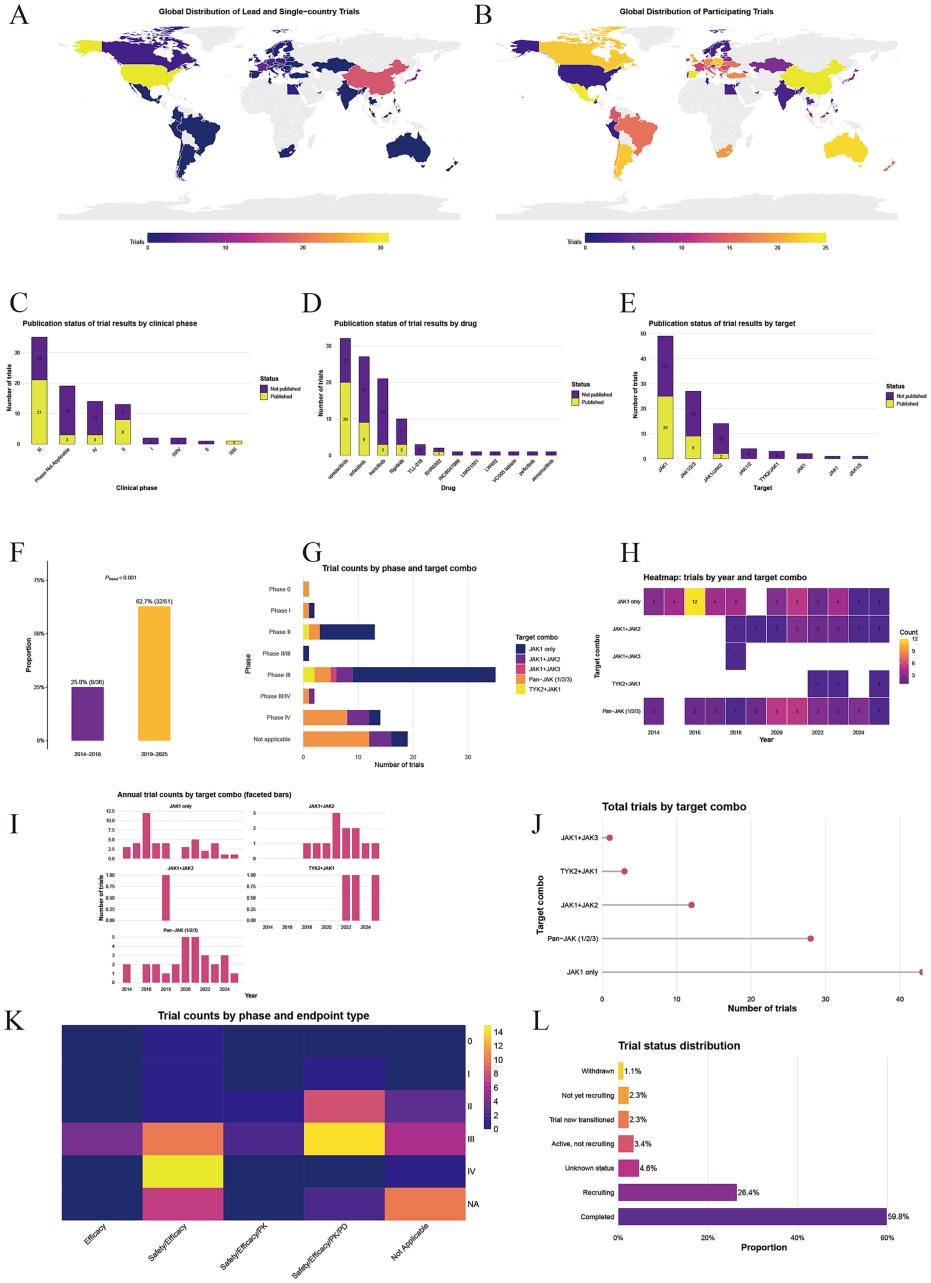
Comprehensive analysis of clinical trials of JAK kinase inhibitors for the treatment of rheumatoid arthritis. **(A)** Distribution of clinical trials by lead country and single countries. **(B)** Distribution of clinical trials in participating countries. **(C)** Publication bias in the experimental phase. **(D)** Publication bias of drugs. **(E)** Publication bias of targets. **(F)** Temporal dimension statistics of target bias. **(G)** Heatmap of target year relationships. **(H)** Target stage relationship diagram. **(I)** Target time relationship histogram. **(J)** Number of targets. **(K)** Heatmap of the relationship between trial phase and endpoint type. **(L)** Experimental status statistics chart.

**Table 1:** Distribution of experiments across the country.

Country	Lead	Single-country	Participating	Total
China	3	14	25	42
United States	25	6	2	32
Spain	0	0	25	25
Poland	3	0	22	25
Hungary	0	0	23	23
Mexico	0	0	24	24
Australia	0	0	23	23
Belgium	0	0	21	21
Argentina	0	0	22	22
Canada	0	3	22	25
Germany	0	4	18	22
Russia	0	0	22	22
Czech Republic	0	0	22	22
United Kingdom	0	2	20	22
South Korea	0	1	19	20
Israel	0	0	21	21
Chile	0	0	20	20
South Africa	0	1	19	20
Turkey	0	0	18	18
Bulgaria	1	0	17	18
Slovakia	0	0	17	17
Ukraine	0	0	16	16
Japan	2	6	8	16
Italy	0	1	15	16
Brazil	0	0	16	16
New Zealand	0	0	16	16
France	1	5	12	18
Serbia	0	0	13	13
Romania	0	0	13	13
Colombia	0	0	14	14
Greece	0	1	11	12
Portugal	0	0	10	10
Switzerland	0	0	10	10
Ireland	0	0	10	10
Latvia	0	0	9	9
Belarus	0	0	8	8
Netherlands	0	0	9	9
Malaysia	0	0	8	8
Kazakhstan	0	0	7	7
Bosnia and Herzegovina	0	0	7	7
Austria	0	0	6	6
Estonia	0	0	6	6
European Union	0	0	6	6
Croatia	0	0	6	6
Lithuania	0	0	5	5
Sweden	0	1	3	4
Georgia	0	0	4	4
Thailand	0	0	5	5
Denmark	0	0	3	3
India	0	0	3	3
Egypt	0	2	3	5
Singapore	0	0	3	3
Finland	0	1	3	4
Philippines	2	0	1	3
Norway	0	0	2	2
Moldova	0	0	2	2
Serbia and Montenegro	0	0	2	2
Bangladesh	0	1	0	1
Slovenia	0	0	1	1
Guatemala	0	0	1	1
Tunisia	0	0	1	1
North Korea	0	0	1	1
Réunion	0	0	1	1
Peru	0	0	2	2
Puerto Rico	0	0	19	19
United Arab Emirates	0	1	0	1
Monaco	0	0	1	1
Jordan	0	0	1	1
Lebanon	0	0	1	1

**Table 2:** Distribution of experiments in the region.

Region	Lead	Participating	Single-country
North America	25	24	9
Europe	9	370	39
Asia–Pacific	9	175	23
Latin America & Caribbean	0	141	3
Other	0	65	6

North America: United States, Canada.

Europe: Spain, Poland, Hungary, Belgium, Germany, Russia, Czech Republic (Czechia), United Kingdom, Bulgaria, Slovakia, Ukraine, Italy, France, Serbia, Romania, Greece, Portugal, Switzerland, Ireland, Latvia, Belarus, Netherlands, Bosnia and Herzegovina, Austria, Estonia, Croatia, Lithuania, Sweden, Georgia, Denmark, Finland, Norway, Moldova (Republic of Moldova), Slovenia, Serbia and Montenegro, European Union (EU).

Asia–Pacific (Asia and Oceania): China, South Korea, Japan, Malaysia, Kazakhstan, India, Thailand, Singapore, Philippines, Bangladesh, Australia, New Zealand, North Korea.

Latin America and the Caribbean: Argentina, Brazil, Chile, Mexico, Colombia, Guatemala, Peru, Puerto Rico.

Other (Africa and Middle East, etc.): South Africa, Israel, Egypt, Tunisia, Réunion.

The distribution of three roles—leadership, single-country conduct, and collaborative participation—across countries clearly reflects the allocation of global RA drug development resources and differences in clinical needs. By total trial number, China ranked first with 42 trials: 14 single-country trials (indicating a targeted response of domestic R&D to RA clinical needs), 25 participations in multinational collaborative trials (indicating deep integration into international collaboration), and 3 lead trials (positioning it as a representative emerging economy combining “independent research + international collaboration”). The United States followed with 32 trials, with a core strength in leadership (25 lead trials, constituting an absolute majority of global lead trials, underscoring its leading position in JAK inhibitor development), supplemented by 6 single-country trials (focused on efficacy verification in specific domestic populations), and participation in only 2 collaborative trials (reflecting a role centered on “leading R&D” rather than “passive participation”).

In addition, Spain and Poland each had 25 trials: Spain participated exclusively in collaborative trials (25), serving as a key clinical site in European multinational studies; Poland combined 3 lead trials and 22 participations, becoming an important hub linking Western European R&D with Eastern European clinical resources in Central and Eastern Europe. Hungary (23 trials) and Mexico (24 trials) mainly acted as collaborative participants (23 and 24 trials, respectively), serving as core platforms for hosting international multicenter trials in Central Europe and in North America/Latin America, covering diverse regional patient populations. Australia (23 trials), Belgium (21 trials), Argentina (22 trials), Canada (25 trials), Germany (22 trials), Russia (22 trials) and the Czech Republic (22 trials) primarily participated in collaborative trials, collectively providing key support to the global clinical trial network. Among them, Germany (4 single-country trials), France (5 single-country trials) and Japan (6 single-country trials) also conducted a number of single-country trials, targeting disease characteristics of local RA populations (e.g., race-specific drug metabolism in Japan; comorbidity distributions in Germany). Notably, France also led 1 trial, while Japan led 2 trials, demonstrating their dual focus on localized verification and limited independent leadership. The Philippines, as a representative of Southeast Asia, led 2 trials and participated in 1 collaborative trial (totaling 3 trials), providing an important supplement to regional R&D participation.

Other countries and regions also contributed to the global clinical trial network with differentiated participation modes: South Korea, South Africa, the United Kingdom, Italy, Greece, Sweden, Finland, Egypt, and the United Arab Emirates conducted a small number of single-country trials while engaging in collaborative projects, balancing localized research needs with international integration. Countries including the United Kingdom (20 collaborative trials), South Korea (19 collaborative trials), and Israel (21 collaborative trials) served as important nodes in regional collaborative networks. Smaller economies such as Latvia (9 trials), Belarus (8 trials), and Malaysia (8 trials) mainly participated in collaborative trials, expanding the geographical coverage of clinical data collection.

These data indicate that global clinical trials of JAK inhibitors for RA have formed a pattern of “the United States leading the R&D direction, China combining autonomy and collaboration, Europe building a multinational collaborative network, and Latin America and other Asia–Pacific countries providing clinical resources.” This structure preserves Western countries’ traditional advantages in R&D design and, through differentiated participation by countries such as China, Poland, and the Philippines, covers broader geographical populations and clinical settings, providing more comprehensive evidence for the safe and effective use of these drugs across different healthcare environments and racial backgrounds.

### Publication bias analysis (Figures 2C–E)

There is significant selective publication bias in the disclosure of trial results for JAK inhibitors in rheumatoid arthritis. At the drug level, mature agents such as upadacitinib and tofacitinib have far more disclosed results than investigational drugs such as INCBO47986 and zemprocitinib. At the target level, disclosures for JAK1-targeted trials (*n* = 25) markedly exceed those for multi-target or novel targets such as JAK1/JAK2 and TYK2/JAK1. At the trial phase level, phase III results (*n* = 21) predominate, whereas phases I and IV have almost no valid results.

### Temporal evolution analysis of target strategies for JAK inhibitor development in RA (Figures 2F,G,I)

The target strategy for JAK inhibitor development in RA shows marked temporal iteration: a test for trend in proportions confirmed that the proportion of clinical trials targeting JAK2 or JAK3 in 2019–2025 (62.7%) was significantly higher than in 2014–2018 (25%) (*p* < 0.01). The heatmap further depicted the temporal distribution of target combinations, with early development centered on concentrated single-target JAK1 trials (12 in 2016), followed by a gradual transition toward JAK2/JAK3 targets and a TYK2 + JAK1 multi-target precise synergy model, while Pan-JAK trials showed a declining proportion.

### Distribution of trial phases and target combinations in clinical trials of JAK inhibitors for rheumatoid arthritis (Figure 2H)

In phase III trials, JAK1-only (JAK1 only) predominates (more than 20 trials). In phase II trials, both Pan-JAK and JAK1-only have notable proportions. Phase I and phase 0 trials are mainly Pan-JAK. Multi-target combinations such as JAK1 + JAK2, JAK1 + JAK3, and TYK2 + JAK1 have low proportions across phases. Notably, this figure presents only target composition by phase, not drug evolution.

### Analysis of target combinations, trial phase–endpoint design, and study status in clinical trials of JAK inhibitors for rheumatoid arthritis (Figures 2J–L)

From the perspective of target combinations, the number of JAK1 single-target trials (>40) is markedly higher than that of Pan-JAK and multi-target combinations (e.g., JAK1 + JAK2, TYK2 + JAK1), underscoring the central role of JAK1 single targeting in development. By trial phase and endpoint type, phase III trials mainly adopted composite endpoints of Safety/Efficacy/Pharmacokinetics/Pharmacodynamics (14 trials), phase II also emphasized such endpoints, whereas phase I focused on basic endpoints, reflecting phase-specific alignment between trial phase and endpoint design. By study status, 59.8% of trials were completed and 26.4% were recruiting, indicating substantial accumulation of research outputs with ongoing projects continuing to advance. Overall, these data reveal a pattern in which JAK inhibitor development centers on JAK1 single targeting, later-phase trials emphasize verification using composite endpoints, and the translation of research outputs is progressing well.

### Efficacy and safety (Tables 3, 4)

**Table 3:** Safety and efficacy of JAK Inhibitor.

Trial ID	Phase	Clinical population	Control arm drug	Experimental arm drug	Target	Assessment timepoint	ACR20 (control/experimental)	ACR50 (control/experimental)	ACR70 (control/experimental)	SAEs (control/experimental)	Non-SAEs (control/experimental)
NCT02629159	Phase III	Adults with insufficient response to methotrexate	Placebo	15 mg upadacitinib	JAK1	12 weeks	36.4 (32.7 to 40.1)/70.5 (67.0 to 74.0)	14.9 (12.2 to 17.6)/45.2 (41.3 to 49.0)	4.9 (3.3 to 6.6)/24.9 (21.6 to 28.2)	2.91%/4.28%	6.60%/4.89%
NCT02889796	Phase III	Adults with insufficient response to methotrexate	Placebo	200 mg Filgotinib	JAK1	12 weeks	49.9 (45.3 to 54.5)/76.6 (72.7 to 80.5)	19.8 (16.1 to 23.5)/47.2 (42.6 to 51.8)	6.7 (4.4 to 9.1)/26.1 (22.1 to 30.2)	4.42%/7.37%	12.84%/26.95%
NCT02706951	Phase III	Adults with insufficient response to methotrexate	25 mg Methotrexate	15 mg Upadacitinib	JAK1	14 weeks	41.2 (34.6 to 47.8)/67.7 (61.5 to 74.0)	15.3 (10.5 to 20.1)/41.9 (35.4 to 48.5)	2.8 (0.6 to 5.0)/22.6 (17.0 to 28.1)	3.24%/5.07%	22.69%/19.82%
NCT05572567	Phase not applicable	Adult patients with rheumatoid arthritis	Methotrexate	Tofacitinib	JAK1/2/3	330 weeks	47.1 (41.2 to 53.0)/44.4 (37.6 to 51.2)	35.9 (30.2 to 41.5)/33.2 (26.7 to 39.6)	21.4 (16.5 to 26.2)/22.4 (16.7 to 28.1)	—	—
NCT02720523	Phase II/III	Patients with insufficient response to bDMARD treatment	Placebo	30 mg Upadacitinib	JAK1	12 weeks	42.9 (29.0 to 56.7)/80.0 (68.9 to 91.1)	16.3 (6.0 to 26.7)/58.0 (44.3 to 71.7)	2.0 (0.0 to 6.0)/28.0 (15.6 to 40.4)	0%/10.00%	22.45%/30.61%
NCT02873936	Phase III	Adult patients with insufficient response to bDMARD treatment	Placebo	100 mg Filgotinib	JAK1	12 weeks	31.1 (23.3 to 38.9)/57.5 (49.4 to 65.7)	14.9 (8.8 to 20.9)/32.0 (24.3 to 39.7)	6.8 (2.4 to 11.1)/14.4 (8.5 to 20.3)	3.38%/5.23%	20.95%/22.88%
NCT03773978	Phase III	Adolescents who do not respond adequately to at least one traditional or biological anti-rheumatic drug	Placebo	4 mg Baricitinib	JAK1/2	44 weeks	—	37 (26.5 to 47.6)/63.4 (53 to 73.8)	35.8 (25.4 to 46.2)/53.7 (42.9 to 64.5)	3.70%/4.88%	12.35%/31.71%
NCT02265705	Phase III	Patients with moderate to severe active rheumatoid arthritis who do not respond well to methotrexate treatment	Placebo	4 mg Baricitinib	JAK1/2	12 weeks	28.3/58.6	—	—	2.76%/2.76%	49.66%/64.83%

**Table 4:** Common adverse events and serious adverse events of JAK inhibitor [affected/at risk (%)].

Trial registration number	Phase	JAK inhibitors	Common adverse events	Serious adverse events (Grade ≥3)
NCT02629159 26 weeks	Phase III	Upadacitinib	Infections and infestations (11.23%)	Infections and infestations (2.31%), injury, poisoning and procedural complications (0.75%), respiratory, thoracic and mediastinal disorders (0.45%), hepatobiliary disorders (0.30%), nervous system disorders (0.30%), cataract (0.15%), gastrointestinal disorders (0.15%)
NCT02886728 52 weeks	Phase III	Filgotinib	Infections and infestations (21.91%), gastrointestinal disorders (7.14%), hypertension (7.14%), headache (3.81%), alopecia (1.90%), increased alanine aminotransferase (1.43%)	Infections and infestations (2.4%), musculoskeletal and connective tissue disorders (1.44%), general disorders (0.96%), gastrointestinal disorders (0.96%), haematological and lymphatic system disorders (0.48%), cardiac disorders (0.48%), prurigo (0.48%), nervous system disorders (0.48%), renal and urinary system disorders (0.48%)
NCT02831855 52 weeks	Phase IV	Tofacitinib	Infections and infestations (4.55%), musculoskeletal and connective tissue disorders (2.65%), increased alanine aminotransferase (1.89%), increased aspartate aminotransferase (1.89%)	Infections and infestations (0.76%), injury, poisoning and procedural complications (0.76%), benign, malignant and unspecified neoplasms (including cysts and polyps) (0.76%), respiratory, thoracic and mediastinal disorders (0.76%), gastrointestinal disorders (0.38%), musculoskeletal and connective tissue disorders (0.38%), nervous system disorders (0.38%), renal and urinary system disorders (0.38%)
NCT03773978 341 days	Phase III	Baricitinib	Infections and infestations (18.3%), nervous system disorders (9.76%), musculoskeletal and connective tissue disorders (7.32%), respiratory, thoracic and mediastinal disorders (6.10%)	Infections and infestations (2.44%), nervous system disorders (1.22%), respiratory, thoracic and mediastinal disorders (1.22%)

Due to the limited disclosure of detailed adverse event classification data in some included trials (e.g., incomplete reporting of CAE subtypes or lack of stratified SAE data), it is not feasible to extend this table to all 87 trials. The four selected trials are representative of the core approved JAK inhibitors for RA, with comprehensive and standardized safety data disclosure, aiming to provide a concise and comparable overview of the safety profiles of different target-specific JAK inhibitors.

Selective JAK1 inhibitors (e.g., upadacitinib, filgotinib) showed significant advantages in short-term (12–14 weeks) efficacy assessments in specific clinical populations. For instance, upadacitinib in the NCT02629159 trial, which enrolled adults with insufficient response to methotrexate (MTX), had an ACR20 rate of 36.4% in the placebo control group and 70.5% in the 15 mg upadacitinib experimental group at week 12. Similarly, filgotinib in the NCT02889796 trial (target population: adults with insufficient response to MTX) achieved an ACR20 rate of 49.9% in the placebo control group and 76.6% in the 200 mg filgotinib experimental group at the same time point. Additionally, upadacitinib also demonstrated efficacy in patients with insufficient response to biological disease-modifying antirheumatic drugs (bDMARDs): the NCT02720523 trial (phase II/III) showed an ACR20 rate of 42.9% in the placebo control group and 80.0% in the 30 mg upadacitinib experimental group at week 12. Filgotinib also had favorable performance in this bDMARD-failure population, as the NCT02873936 trial (phase III) reported an ACR20 rate of 31.1% in the placebo control group and 57.5% in the 100 mg filgotinib experimental group at week 12.

The JAK1/2 inhibitor baricitinib exhibited sustained and stable long-term efficacy in the NCT03773978 trial, which included adolescents who did not respond adequately to at least one traditional or biological anti-rheumatic drug. After 44 weeks of follow-up, the ACR50 rate was 37% in the placebo control group and 63.4% in the 4 mg baricitinib experimental group, while the ACR70 rate was 35.8% in the placebo control group and 53.7% in the experimental group. In another phase III trial (NCT02265705) targeting adults with moderate to severe active rheumatoid arthritis who responded poorly to MTX, baricitinib (4 mg) achieved an ACR20 rate of 58.6% at week 12, significantly higher than the 28.3% in the placebo control group.

The pan-JAK inhibitor tofacitinib was evaluated in a 330-week long-term trial (NCT05572567) involving adult patients with rheumatoid arthritis, with MTX as the active control. The ACR20 rate was 47.1% in the MTX control group and 44.4% in the tofacitinib experimental group, indicating that the efficacy tended to plateau over the long term.

In terms of safety, serious adverse event (SAE) rates differed among drugs and populations. In the MTX-insufficient response population: upadacitinib (NCT02629159) had SAE rates of 2.91% in the placebo control group and 4.28% in the experimental group; filgotinib (NCT02889796) had SAE rates of 4.42% in the placebo control group and 7.37% in the experimental group; another upadacitinib trial (NCT02706951, active control with 25 mg MTX) reported SAE rates of 3.24% in the MTX group and 5.07% in the 15 mg upadacitinib group. In the bDMARD-failure population: upadacitinib (NCT02720523) had 0% SAEs in the placebo group and 10.00% in the experimental group; filgotinib (NCT02873936) had SAE rates of 3.38% in the placebo group and 5.23% in the experimental group. For baricitinib (NCT03773978) in adolescents with insufficient response to traditional or biological drugs, SAE rates were 3.70% in the placebo group and 4.88% in the experimental group, while non-serious adverse event (non-SAE) rates were 12.35 and 31.71%, respectively, suggesting the need to pay attention to differences in the spectrum of non-serious adverse events for specific drugs. Additionally, baricitinib (NCT02265705) in MTX-poor response adults had non-SAE rates of 49.66% in the placebo group and 64.83% in the experimental group.

Common adverse events (CAEs) and grade ≥3 serious adverse events (SAEs) differed clearly across JAK inhibitor targets: upadacitinib (NCT02629159, JAK1) CAEs were dominated by infections and infestations (11.23%); SAEs involved infections and infestations (2.31%) and injury, poisoning and procedural complications (0.75%) across multiple systems, with each SAE type below 2.5%, and no cardiovascular-related or malignancy-related adverse events were recorded. Filgotinib (NCT02886728, JAK1) had the highest CAE rate in infections and infestations (21.91%), accompanied by gastrointestinal disorders (7.14%) and hypertension (7.14%, a cardiovascular-related adverse event); SAEs were mainly infections and infestations (2.4%) and musculoskeletal and connective tissue disorders (1.44%), with cardiac disorders (a cardiovascular-related SAE) occurring at a low rate of 0.48%, and no malignancy-related adverse events detected. Tofacitinib (NCT02831855, JAK1/2/3) had overall lower CAE rates, with infections and infestations (4.55%) and musculoskeletal and connective tissue disorders (2.65%) as the main CAEs; among SAEs, benign, malignant and unspecified neoplasms (including cysts and polyps, a malignancy-related event) occurred at 0.76%, while no cardiovascular-related CAEs or SAEs other than the above-mentioned categories were observed, and other SAE types (infections and infestations; injury, poisoning and procedural complications, etc.) were in the range of 0.38–0.76%. Baricitinib (NCT03773978, JAK1/2) CAEs were mainly infections and infestations (18.3%) and nervous system disorders (9.76%); SAEs were concentrated in infections and infestations (2.44%), nervous system disorders (1.22%), and respiratory, thoracic and mediastinal disorders (1.22%), with no cardiovascular or malignancy-related adverse events reported.

The tumor and cardiovascular-related adverse events associated with JAK inhibitors in the treatment of immune-mediated inflammatory diseases have been reported by relevant experts’ consensus and systematic reviews (7, 8). In the four analyzed trials, fingolimod (NCT02886728) showed adverse events in the cardiovascular system, manifested as hypertension (7.14%, CAE) and heart diseases (0.48%, SAE), with a relatively low incidence rate; while in the case of malignant tumors, adverse events were found in tofacitinib (NCT02831855), with the SAE incidence rate of benign, malignant and unspecified tumors being 0.76%. During the observation period, upadacitinib (NCT02629159) and baricitinib (NCT03773978) did not show safety signals in the cardiovascular or malignant tumor aspects. The reason for this might be that the time when adverse events were reported for upadacitinib (26 weeks), baricitinib (341 days), was longer than that for tofacitinib and fingolimod (both 52 weeks).

## Discussion

This study, based on 87 clinical trials registered across 16 platforms from 2014–2025, delineates the global evidence structure and R&D evolution of JAK inhibitors in the treatment of rheumatoid arthritis (RA). Overall, JAK1 single-target agents dominate both in trial volume and in result disclosure. Phase III studies more often employ composite endpoints of efficacy, safety, and pharmacokinetics/pharmacodynamics (PK/PD), phase I focuses on safety and pharmacokinetics, and phase II emphasizes dose–response exploration. Over time, the past five years have seen a gradual increase in trials targeting JAK2, JAK3, and multi-target combinations (e.g., JAK1 + TYK2), with a decline in the proportion of Pan-JAK trials. This trend reflects attention to target selectivity and risk–benefit rebalancing, partly attributable to clinical warnings from adverse signals (cardiovascular, malignancy) revealed by ORAL Surveillance and other large observational studies (9).

In terms of efficacy and adverse events, data support that JAK inhibitors provide superior short- to mid-term clinical responses compared with cDMARDs; some agents (e.g., upadacitinib) show efficacy comparable to mainstream bDMARDs (e.g., TNF-*α* inhibitors) with divergent safety profiles (NCT02706951). For example, in the SELECT-EARLY trial of upadacitinib, the week-12 ACR20 reached 70.5%, significantly higher than the control group (36.4%) (NCT02629159). Filgotinib likewise demonstrated favorable efficacy in patients with prior treatment failure in FINCH 1 (ACR20 76.6% vs. 49.9%) (NCT02889796). Baricitinib showed sustained efficacy advantages in long-term follow-up of the RA-BEAM trial (NCT03773978, 44 weeks). However, regarding long-term safety, ultra-long follow-up of tofacitinib and other agents (>6 years) indicates that efficacy metrics such as ACR20 tend to plateau (NCT05572567, 330 weeks), and EULAR has explicitly indicated increased signals of cardiovascular events, thrombosis, and malignancy in high-risk populations (10).

At the regional level, under a global multicenter collaboration model, China, the United States, Europe, and the Asia-Pacific are complementary in study leadership and patient sources, but except for a few trials, prespecified regional subgroup analyses and interaction testing are extremely limited, which weakens the regional comparability of evidence generalization (11, 12). In current registration studies, for both endpoint types and safety-event reporting, stratified analyses by regional/national healthcare environments, ethnic differences, and care pathways are not performed, constituting a potential blind spot in evidence applicability against the backdrop of high multinational participation (13).

The target strategy for JAK inhibitor development in RA exhibits temporal iteration highly aligned with disciplinary understanding and clinical needs, providing key reference for subsequent drug development and clinical application. A trend test for proportions confirmed that the share of clinical trials targeting JAK2 or JAK3 rose to 62.7% in 2019–2025 (vs. 25% in 2014–2018, *p* < 0.01). Together with heatmap-presented temporal changes in target combinations, this indicates a shift from an early exploration phase centered on JAK1 single-target studies (12 trials in 2016) toward JAK2/JAK3 targets and TYK2 + JAK1. This transition is not random but based on deeper understanding of RA pathogenesis and dynamic adjustment to clinical needs. The early focus on JAK1 single targeting derived from the field’s initial recognition of JAK isoform functions—JAK1 as a key isoform mediating signals of core pro-inflammatory factors such as IL-6 in RA—where single-target inhibition could rapidly fill therapeutic gaps in patients with inadequate response to methotrexate or biologics while reducing early development risk (14). The subsequent shift to specific isoform combinations reflects both a response to individualized clinical needs and consolidation of mechanistic insights; for example, aberrant activation of JAK2 exacerbates joint bone destruction (15), JAK3 predominantly governs lymphocyte activation (16), and TYK2 regulates type I IFN–related chronic inflammation (17). Multi-target synergy can cover patient subgroups with activation of different inflammatory pathways, overcoming response limitations of single-target drugs, whereas pan-JAK inhibitors, lacking isoform selectivity, readily induce off-target adverse reactions such as infections, making their risk–benefit ratio less compatible with current precision treatment demands in RA and leading to their gradual retreat from mainstream development. This shift from single-target exploration to multi-target precise synergy not only reflects an upgrade from “broad-spectrum inhibition” to “precise modulation,” but, by tightly linking target selection with inflammatory mechanisms and clinical pain points, also lays an important foundation for optimizing individualized RA treatment and guiding the development of new JAK inhibitors.

Meanwhile, disclosure shows an evident imbalance across the “phase–target” hierarchy. Public results for JAK1-selective molecules, phase III, and mature drugs are relatively sufficient, whereas disclosure for new targets and post-marketing (phase IV) studies is seriously inadequate: in the dataset, only seven trials were strictly labeled as phase IV, accounting for just 16.09%. This limits real-world risk assessment of rare and delayed safety events (e.g., MACE, VTE, malignancy) (9). In addition, updated EULAR guidelines recommend cautious use of JAK inhibitors in high-risk patients (10).

Based on the above structural evidence features, future research requires targeted optimization. First, JAK inhibitors on the market for more than ten years (e.g., tofacitinib, baricitinib) should accelerate high-quality, long-term phase IV studies, focusing on high-risk populations (e.g., older adults, those with prior cardiovascular and thrombotic complications), incorporating functional outcomes and quality-of-life measures as well as practical management strategies such as vaccination and concomitant medications, to improve real-world risk–benefit evaluation. Second, all multinational and multiregional clinical trials should preregister regional stratification or interaction-analysis plans at the design stage, ensure adequate sample coverage and region-stratified interpretability, and enhance global applicability of data. Third, balanced comparisons of non-JAK1-selective agents (e.g., JAK2, JAK3, TYK2 alone or in combination) versus JAK1 agents should be expedited through head-to-head or platform trials, with harmonized primary efficacy endpoints (e.g., ACR50, DAS28-CRP low disease activity attainment) and safety outcomes, to mitigate target-effect selection bias arising from uneven disclosure. In addition, all studies should commit to staged and time-limited disclosure of key data, and incorporate pharmacodynamic biomarkers and digital patient-reported outcomes to advance molecularly precise prescribing and risk-mitigation pathways. In sum, the global evidence landscape for JAK inhibitors still has key gaps; only by taking registration data as a starting point, specifically addressing post-marketing evidence and regional subgroup-validation shortfalls, and advancing target diversity and transparent comparisons can overall optimization of safety and access be achieved in clinical practice, guidelines, and reimbursement decision-making.

Although this study systematically organized the global clinical trial landscape of JAK inhibitors for RA from 2014 to 2025, clearly presenting target-evolution patterns, regional participation features, and differences in drug efficacy and safety to inform clinical practice and R&D, the aforementioned limitations must be acknowledged—publication bias leading to overconcentration of evidence on mature drugs/targets/phase III trials, lack of a Cochrane framework for systematic trial-quality assessment, absence of stratified analyses for geographic heterogeneity, and inability to extract reasons for trial termination from original data—all of which restrict the completeness, credibility, and regional generalizability of the conclusions. Subsequent research could reduce publication bias by promoting standardized disclosure of clinical-trial data, introduce Cochrane quality-assessment systems to verify trial rigor, prespecify regional subgroup-analysis plans in multicenter trials to match diverse healthcare settings, and call on registries to supplement key information such as reasons for trial termination. Only by addressing these evidence gaps can the true value of JAK inhibitors in RA treatment be interpreted more objectively and comprehensively, providing stronger evidence to optimize clinical decision-making, update guidelines, and develop new JAK inhibitors, ultimately improving treatment benefits and quality of life for patients with RA. Despite the aforementioned limitations, this study has several novelties: (1) It systematically analyses 87 global clinical trials of JAK inhibitors for RA from 2014 to 2025, covering 16 databases/registries, providing a comprehensive and up-to-date global landscape of R&D, regional participation, and efficacy/safety outcomes. (2) It reveals the temporal evolution of target strategies (from single JAK1 to JAK2/JAK3 and multi-target combinations) and quantifies the publication bias across drugs, targets, and trial phases, which has not been fully addressed in previous studies. (3) It clarifies the stratified and complementary regional participation pattern (U.S. leading R&D, China combining autonomy and collaboration, Europe building multinational networks) and provides targeted suggestions for optimizing subsequent trials (e.g., regional subgroup analysis, long-term phase IV studies), offering valuable references for clinical practice and drug R&D.

## Data Availability

The original contributions presented in the study are included in the article/Supplementary material, further inquiries can be directed to the corresponding authors.

## Supplementary material

APPENDIX TABLE 1 Clinical trials included in the study.
