# Supplementary material for: Application of JAK inhibitors in the treatment of rheumatoid arthritis: a systematic analysis based on clinical trial databases and registries
Source: Front Med (Lausanne). 2026 Mar 5;13:1680115. doi: 10.3389/fmed.2026.1680115 (PMC12999776; doi:10.3389/fmed.2026.1680115)
Supplement: APPENDIX TABLE 1 — Clinical trials included in the study. [file Supplementary_file_1.pdf]

1.China Clinical Trial Registration Center
研究疾病名称:类风湿关节炎
药物名称:JAK 抑制剂

2.China Drug Clinical Trial Registration and Information Disclosure Platform

适应症:类风湿关节炎
药物名称:JAK 抑制剂

3.U.S.Clinical Trial Registration PlatformIran Clinical Trial Registration Platform
(‘Arthritis, Rheumatoid’ OR ‘rheumatism arthritis’ OR ‘rheumatoid arthritis’) AND (‘Inhibitors, Janus Kinase’ OR ‘Kinase Inhibitors, Janus’ OR ‘Janus Kinase Inhibitor’ OR ‘Inhibitor, Janus Kinase’ OR ‘Kinase Inhibitor, Janus’ OR ‘JAK Inhibitor’ OR ‘Inhibitor, JAK’ OR ‘JAK Inhibitors’ OR ‘Inhibitors, JAK’)

4.**UK Clinical Trial Registration Platform**

(‘Arthritis, Rheumatoid’ OR ‘rheumatism arthritis’ OR ‘rheumatoid arthritis’) AND (‘Inhibitors, Janus Kinase’ OR ‘Kinase Inhibitors, Janus’ OR ‘Janus Kinase Inhibitor’ OR ‘Inhibitor, Janus Kinase’ OR ‘Kinase Inhibitor, Janus’ OR ‘JAK Inhibitor’ OR ‘Inhibitor, JAK’ OR ‘JAK Inhibitors’ OR ‘Inhibitors, JAK’)

5.EU Clinical Trials Register
(‘Arthritis, Rheumatoid’ OR ‘rheumatism arthritis’ OR ‘rheumatoid arthritis’) AND (‘Inhibitors, Janus Kinase’ OR ‘Kinase Inhibitors, Janus’ OR ‘Janus Kinase Inhibitor’ OR ‘Inhibitor, Janus Kinase’ OR ‘Kinase Inhibitor, Janus’ OR ‘JAK Inhibitor’ OR ‘Inhibitor, JAK’ OR ‘JAK Inhibitors’ OR ‘Inhibitors, JAK’)

6.India Clinical Trial Registration Platform
Arthritis, Rheumatoid;rheumatism arthritis;rheumatoid arthritis;Inhibitors, Janus Kinase;Kinase Inhibitors, Janus;Janus Kinase Inhibitor;Inhibitor, Janus Kinase;Kinase Inhibitor, Janus;JAK Inhibitor;Inhibitor, JAK;JAK Inhibitors;Inhibitors, JAK

7.Hong Kong Clinical Trial Registration Platform
(‘Arthritis, Rheumatoid’ OR ‘rheumatism arthritis’ OR ‘rheumatoid arthritis’) AND (‘Inhibitors, Janus Kinase’ OR ‘Kinase Inhibitors, Janus’ OR ‘Janus Kinase Inhibitor’ OR ‘Inhibitor, Janus Kinase’ OR ‘Kinase Inhibitor, Janus’ OR ‘JAK Inhibitor’ OR ‘Inhibitor, JAK’ OR ‘JAK Inhibitors’ OR ‘Inhibitors, JAK’)

8.Korea Clinical Trial Registration Platform
(‘Arthritis, Rheumatoid’ OR ‘rheumatism arthritis’ OR ‘rheumatoid arthritis’) AND (‘Inhibitors, Janus Kinase’ OR ‘Kinase Inhibitors, Janus’ OR ‘Janus Kinase Inhibitor’ OR ‘Inhibitor, Janus Kinase’ OR ‘Kinase Inhibitor, Janus’ OR ‘JAK Inhibitor’ OR ‘Inhibitor, JAK’ OR ‘JAK Inhibitors’ OR ‘Inhibitors, JAK’)

9. Japan Clinical Trial Registration Platform

Name of the target (disease)： Arthritis, Rheumatoid rheumatism arthritis rheumatoid arthritis

Name of the target (Medication): Inhibitors, Janus Kinase Kinase Inhibitors, Janus Janus Kinase Inhibitor Inhibitor, Janus Kinase Kinase Inhibitor, Janus JAK Inhibitor Inhibitor, JAK JAK Inhibitors Inhibitors, JAK

10. Germany Clinical Trial Registration Platform

('Arthritis, Rheumatoid' OR 'rheumatism arthritis' OR 'rheumatoid arthritis') AND ('Inhibitors, Janus Kinase' OR 'Kinase Inhibitors, Janus' OR 'Janus Kinase Inhibitor' OR 'Inhibitor, Janus Kinase' OR 'Kinase Inhibitor, Janus' OR 'JAK Inhibitor' OR 'Inhibitor, JAK' OR 'JAK Inhibitors' OR 'Inhibitors, JAK')

11. Iran Clinical Trial Registration Platform

('Arthritis, Rheumatoid' OR 'rheumatism arthritis' OR 'rheumatoid arthritis') AND ('Inhibitors, Janus Kinase' OR 'Kinase Inhibitors, Janus' OR 'Janus Kinase Inhibitor' OR 'Inhibitor, Janus Kinase' OR 'Kinase Inhibitor, Janus' OR 'JAK Inhibitor' OR 'Inhibitor, JAK' OR 'JAK Inhibitors' OR 'Inhibitors, JAK')

12. Brazil Clinical Trial Registration Platform

('Arthritis, Rheumatoid' OR 'rheumatism arthritis' OR 'rheumatoid arthritis') AND ('Inhibitors, Janus Kinase' OR 'Kinase Inhibitors, Janus' OR 'Janus Kinase Inhibitor' OR 'Inhibitor, Janus Kinase' OR 'Kinase Inhibitor, Janus' OR 'JAK Inhibitor' OR 'Inhibitor, JAK' OR 'JAK Inhibitors' OR 'Inhibitors, JAK')

13. Thailand Clinical Trial Registration Platform

('Arthritis, Rheumatoid' OR 'rheumatism arthritis' OR 'rheumatoid arthritis') AND ('Inhibitors, Janus Kinase' OR 'Kinase Inhibitors, Janus' OR 'Janus Kinase Inhibitor' OR 'Inhibitor, Janus Kinase' OR 'Kinase Inhibitor, Janus' OR 'JAK Inhibitor' OR 'Inhibitor, JAK' OR 'JAK Inhibitors' OR 'Inhibitors, JAK')

14. Netherlands Clinical Trial Registration Platform

('Arthritis, Rheumatoid' OR 'rheumatism arthritis' OR 'rheumatoid arthritis') AND ('Inhibitors, Janus Kinase' OR 'Kinase Inhibitors, Janus' OR 'Janus Kinase Inhibitor' OR 'Inhibitor, Janus Kinase' OR 'Kinase Inhibitor, Janus' OR 'JAK Inhibitor' OR 'Inhibitor, JAK' OR 'JAK Inhibitors' OR 'Inhibitors, JAK')

15. Africa Clinical Trial Registration Platform

(‘Arthritis, Rheumatoid’ OR ‘rheumatism arthritis’ OR ‘rheumatoid arthritis’) AND (‘Inhibitors, Janus Kinase’ OR ‘Kinase Inhibitors, Janus’ OR ‘Janus Kinase Inhibitor’ OR ‘Inhibitor, Janus Kinase’ OR ‘Kinase Inhibitor, Janus’ OR ‘JAK Inhibitor’ OR ‘Inhibitor, JAK’ OR ‘JAK Inhibitors’ OR ‘Inhibitors, JAK’)

16. Australia Clinical Trial Registration Platform

(‘Arthritis, Rheumatoid’ OR ‘rheumatism arthritis’ OR ‘rheumatoid arthritis’) AND (‘Inhibitors, Janus Kinase’ OR ‘Kinase Inhibitors, Janus’ OR ‘Janus Kinase Inhibitor’ OR ‘Inhibitor, Janus Kinase’ OR ‘Kinase Inhibitor, Janus’ OR ‘JAK Inhibitor’ OR ‘Inhibitor, JAK’ OR ‘JAK Inhibitors’ OR ‘Inhibitors, JAK’)
